# Polymer Electrolyte Membranes for Water Photo-Electrolysis

**DOI:** 10.3390/membranes7020025

**Published:** 2017-04-29

**Authors:** Antonino S. Aricò, Mariarita Girolamo, Stefania Siracusano, David Sebastian, Vincenzo Baglio, Michael Schuster

**Affiliations:** 1 CNR-ITAE Institute for Advanced Energy Technologies “N. Giordano”, Via Salita S. Lucia sopra Contesse 5, 98126 Messina, Italy; girolamo@itae.cnr.it (M.G.); siracusano@itae.cnr.it (S.S.); sebastian@itae.cnr.it (D.S.); baglio@itae.cnr.it (V.B.); 2 FUMATECH BWT GmbH Gesellschaft für Funktionelle Membranen und Anlagentechnologie mbH, Carl-Benz-Strasse 4, Bietigheim-Bissingen, D-74321 Baden-Württemberg, Germany; schuster@fumatech.de

**Keywords:** anion exchange polymer electrolyte membrane, proton exchange polymer electrolyte membrane, water splitting, photo-electrolysis, TiO_2_, Ti-suboxides

## Abstract

Water-fed photo-electrolysis cells equipped with perfluorosulfonic acid (Nafion^®^ 115) and quaternary ammonium-based (Fumatech^®^ FAA3) ion exchange membranes as separator for hydrogen and oxygen evolution reactions were investigated. Protonic or anionic ionomer dispersions were deposited on the electrodes to extend the interface with the electrolyte. The photo-anode consisted of a large band-gap Ti-oxide semiconductor. The effect of membrane characteristics on the photo-electrochemical conversion of solar energy was investigated for photo-voltage-driven electrolysis cells. Photo-electrolysis cells were also studied for operation under electrical bias-assisted mode. The pH of the membrane/ionomer had a paramount effect on the photo-electrolytic conversion. The anionic membrane showed enhanced performance compared to the Nafion^®^-based cell when just TiO_2_ anatase was used as photo-anode. This was associated with better oxygen evolution kinetics in alkaline conditions compared to acidic environment. However, oxygen evolution kinetics in acidic conditions were significantly enhanced by using a Ti sub-oxide as surface promoter in order to facilitate the adsorption of OH species as precursors of oxygen evolution. However, the same surface promoter appeared to inhibit oxygen evolution in an alkaline environment probably as a consequence of the strong adsorption of OH species on the surface under such conditions. These results show that a proper combination of photo-anode and polymer electrolyte membrane is essential to maximize photo-electrolytic conversion.

## 1. Introduction

The continuous increase of energy consumption and the associated increase of greenhouse gas emissions causing global warming have produced significant concerns. It is widely recognised that there is an urgent need to increase the level of utilisation renewable sources in order to address the environmental issues. Hydrogen is an alternative fuel and, according to its high energy density and clean combustion, can represent a suitable energy vector mediating between renewable sources and sustainable mobility [1,2,3,4]. The energy associated with solar radiation striking the earth is several orders of magnitude the global energy consumption. An efficient and wide-scale use of sunlight can occur through a decentralised conversion into hydrogen that can be stored and used as fuel in several applications including highly efficient fuel cell devices for stationary generation, transportation and portable power [4].

The direct conversion of solar energy to hydrogen represents a long-term objective for the next generation energy system based on renewable sources. In this context, hydrogen generation from photo-electrochemical water splitting using solar energy is of primary interest.

Significant progress has been recently achieved for the photo-electrolytic hydrogen production through the development of advanced water splitting semiconductors. However, this progress remains at the level of laboratory curiosity since relevant aspects dealing with practical development of the photo-electrolysis cell devices, in particular the separator system, have not been addressed with the same efforts. Without a proper development of the technology around the water splitting semiconductors, these systems can hardly became economically competitive with respect to current technologies involving the extraction of fossil fuels and their internal combustion [1,2,3].

As discussed above, most of the efforts made so far have been addressed to the development of new photo-catalytic materials with better efficiencies but often operating under voltage bias-assisted mode. However, to reach the level of commercial viability for the water photo-electrolysis technology, the efforts should also cover the other cell components and provide characterisation in a real device. According to several literature reports [3], it appears that water splitting semiconductors characterised by improved efficiency are often consisting of unstable materials. Some interesting semiconductors showing good electronic properties and proper capability of harvesting the visible fraction of solar radiation have been assessed just in half-cells in the presence of corrosive liquid electrolytes. The latter can produce a chemical attack, thus diminishing the endurance potentialities of the novel materials, whereas solid polymer electrolytes usually stabilise the electrolysis materials against corrosion [5]. It is widely recognised that, in order to further advance the photo-electrolysis technology and favour market diffusion, a parallel development of reliable membrane separators is required. Moreover, the utilisation of appropriate ionomer dispersion at the electrode—electrolyte interface [5,6], providing suitable pH for the photo-electrolysis environment to maximise solar-to-hydrogen-efficiency and offering good stability while being cost-effective, is also relevant. The membrane separator is an important component of electrolysis and photo-electrolysis systems thus avoids recombination of the formed gas products. It provides a pathway for ion conduction, and, together with the ionomer dispersion used in the electrodes, establishes the operating pH of the photo-electrolytic device when this is fed with pure water as largely preferred from a technological viewpoint [3,7]. The membrane and ionomer dispersions are thus part of the advanced device engineering required to maximise the overall performance of the photo-electrolysis system and to prevent corrosion especially in the case of semiconductors with relatively low energy gap [3,7].

A simple configuration for a photo-electrolysis cell consists of a semiconductor photo-anode for the oxygen evolution with relatively large energy gap and a Pt-based counter electrode for hydrogen evolution [8,9]. The photo-anode is illuminated, whereas the Pt cathode operates in the dark. The energy gap of a photo-anode must be larger than that of the energy required for splitting the water molecule into hydrogen and oxygen gases (1.23 eV) and the electronic levels (valence band and conduction band edges) should properly match with the electronic levels associated with the redox couples (half-cell reactions) involved in water splitting [10]. Accordingly, most of the photo-electrolysis cells use large gap semiconductors e.g., TiO_2_, thus absorbing mainly a small UV portion of the solar spectrum [3,7]. This approach does not allow achieving high levels of efficiency and has limited practical interest. However, large band gap TiO_2_ (3.2 eV) has been widely investigated as a model system and can be still used to provide a baseline of comparison when other cell components are developed e.g., alternative cathodes and membranes. The research trend is to use semiconductors with a smaller energy gap in order to harvest a good fraction of the visible region while keeping the band gap above 1.23 eV [3,7]. Other approaches deal with combining the photo-electrolysis cell with a Dye Sensitized Solar Cells (DSSC) [11]. In the latter case, the visible light reaches the DSSC cell and is converted into electricity that can be utilised to help water splitting in the photo-electrolysis cell using the UV fraction of sunlight [11].

TiO_2_ anode semiconductors are characterised by suitable electronic band structure for water splitting in combination with platinum cathodes in the photo-electrolysis cell [3,7,10]. However, the energy gap of 3.2 eV in TiO_2_ limits significantly the level of efficiency in photo-electrochemical systems. Despite this aspect, TiO_2_ represents a model system for photo-electrolysis device being quite stable in all operating conditions and highly photoactive in the specific operating region of the solar spectrum. The wide energy gap also allows the occurrence of a spontaneous photo-voltage [11]. Besides the energy implications, e.g., no need of external electric bias, this aspect can provide insights into the capability of the membrane to separate the formed gas (i.e., direct recombination causing occurrence of mixed potentials is avoided). It is well known that the photo-electrocatalytic properties of titanium dioxide depend on the operating environment, which is similar to most of the water splitting semiconductors [7]. The oxygen evolution process involves a charge transfer between photo-generated carriers at the surface of the TiO_2_ photo-anode and the adsorbed species on the electrode surface, e.g., OH species, which depend very much on the electrolyte properties [3]. The semiconductor/electrolyte interface plays an important role in determining the efficiency and stability of the water photo-electrolysis process [3,7].

This work is aiming at comparing the behaviour of commercial perfluorosulfonic acid (Nafion^®^ 115) and quaternary ammonium-based anionic (Fumatech^®^ FAA3) ion exchange membranes in photo-electrolysis cells fed with pure water. The membrane has the primary role of acting as separator of the hydrogen and oxygen [5,6] evolved at the photo-anode and cathode surface, respectively, and to provide continuity for the transfer of ions (protons or hydroxides) between the two cell compartments. Protonic and alkaline ionomer dispersions (micelles) with the same chemistry of the membranes were used at the cathode and photo-anode to extend the interface between electrodes and electrolyte; these ionomer dispersions contribute together with the membrane to characterize the cell environment in terms of pH and process characteristics (adsorption of active species, ionic conduction-type, ionic conductivity, etc.). High surface area n-type commercial TiO_2_ powder [12] was used as model photo-anode to allow a fair comparison of the membrane characteristics. Modification of the photo-anode surface with a Ti-suboxide layer [13] was also carried out to derive information on the influence of the electrolyte environment on different surface characteristics of the photo-anode for the water splitting process.

## 2. Results and Discussion

### 2.1. Photo-Electrolysis Cell Based on Ion Exchange Membrane Separator

**Figure 1:**
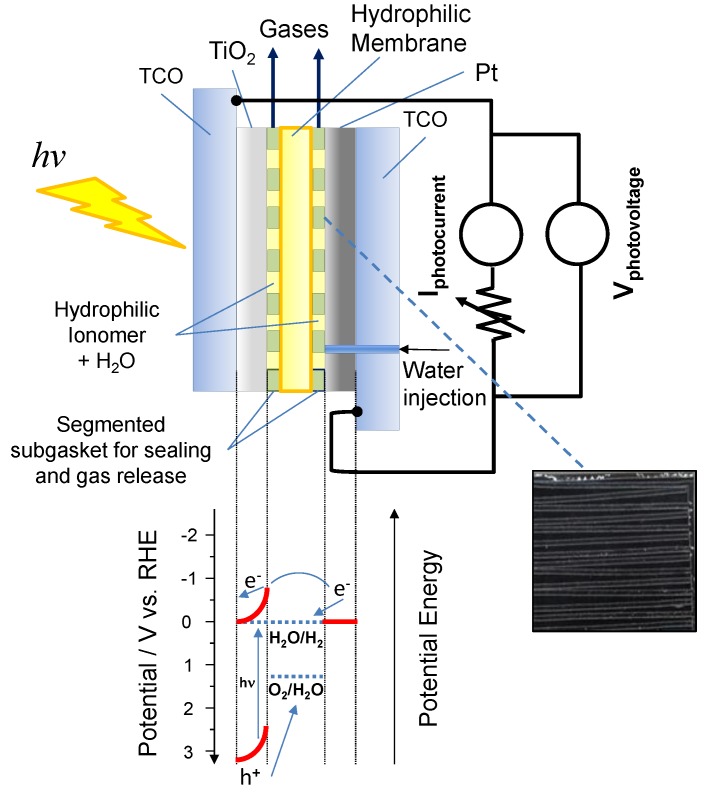
Sketch of the photo-electrolysis cell (top) and energy diagram for the water splitting process under short circuit; the segmented ionically conductive polymer used to favour gas escape and proton percolation is shown on the right; the Fermi levels are equal in the semiconductor and electrolyte (bottom).

A sketch of the photo-electrolysis cell fed with pure deaerated water is represented in Figure 1 together with the energy diagram related to the water splitting process for this device. The cell consists of a glass coated with fluorine doped tin oxide SnO_2_:F forming a transparent conductive oxide (TCO) as support of the TiO_2_ photo-anode (Figure 1, top). The latter is coated with the ionomer dispersion (a solution containing the solid polymer electrolyte micelles, which is deposited on the electrode surface and successively dried to favour micelles adsorption on the electrode layer) to extend the reaction region at the interface and to provide a path for ion percolation. The polymer electrolyte membrane separates the photo-anode from the Pt counter electrode (Figure 1, top). To allow water feed and gas escape from the cell, a segmented sub-gasket is used between the membrane and the electrodes, whereas water is injected through a hole drilled on the backside of the counter electrode. The segmented sub-gasket used between the membrane and the photo-electrode is made of the same ionically conductive polymer as the membrane. This assures the ionic percolation. The free space between each electrode and membrane is filled with water and hydrophilic ion conductive ionomer to favour ionic percolation. The counter electrode is formed by Pt black particles deposited on a TCO.

The principle of operation of the photo-electrolysis cell is also represented in Figure 1 (bottom). Solar irradiation leads to a generation of electron-hole pairs when the wavelength energy is larger than the energy gap of the semiconductor (*hν* > *E*_g_). These pairs are separated by the electric field of the space charge region [3,7]. The holes reaching the semiconductor-electrolyte interface are consumed by the oxygen evolution, whereas electrons, which are forced towards the semiconductor back contact, are transferred, through the external electric circuit, to the Pt cathode where the hydrogen evolution occurs in the dark.

### 2.2. Physico-Chemical Characterization of the Membrane

**Figure 2:**
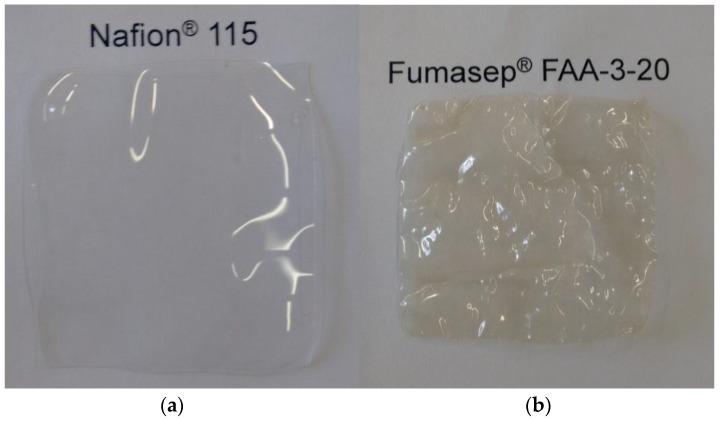
Photographs of Nafion^®^ 115 (**a**) and Fumion^®^ FAA3-20 ionomer membranes (**b**) prior purification.

Commercial perfluorosulfonic acid (Nafion^®^ 115) and quaternary ammonium-based anionic (Fumatech, Fumion Fumasep^®^ FAA-3-20) ion exchange membranes were purified before use (a picture of the as received membranes is shown in Figure 2).

The main properties of the two different membranes used in the present work are presented in Table 1. The nature of polymer chemistry, equivalent weight (EW), membrane thickness, ion conductivity are reported. It is clearly observed that the perfluorosulfonic membrane is characterised by better ionic conductivity than the anionic membrane, despite the fact that equivalent weight is larger for Nafion^®^. Of course, the proton diffusivity is much higher than the diffusivity of hydroxide anions; moreover, the presence of fluorine in the Nafion^®^ backbone enhances dissociation of hydrogen ions of the terminal sulfonic groups in the side chain and the charge separation. Charge separation is also relevant in the anionic membrane due to the ionic character of the bond between terminal ammonium groups and OH^−^. To compensate for the lower conductivity, the anionic membrane was selected significantly thinner than the Nafion^®^ membrane; this was also in light of the fact that the anionic membranes are usually characterised by lower gas cross-over [14].

### 2.3. Photo-Electrode Characterisation

**Figure 3:**
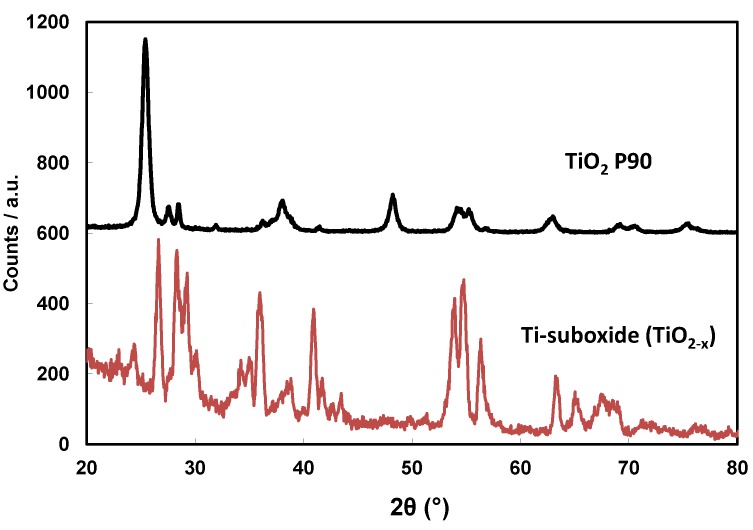
X-ray diffraction patterns of a bare P90 TiO_2_ and Ti-sub-oxide powders used for the photoanode.

According to the X-ray diffraction analysis (Figure 3), the TiO_2_ Degussa P90 powder (Frankfurt, Germany) used as photo-anode precursor mainly showed the presence of an anatase phase. The main characteristic peak at 25.4° 2θ was assigned to the (101) Miller index of anatase (JCPDS schedule: 21-1272) [12,15]. There is a small amount of evidence of the rutile structure (JCPDS schedule: 21-1276) with peaks at 27° (110) and 37° (101) 2θ; the occurrence of small percentages of brookite (JCPDS schedule: 16-617) is not excluded [12]. According to the broadening of the X-ray diffraction peaks, the mean crystallite size for this sample, related to the anatase structure, was about 15 nm as determined from the Scherrer equation. Chemical reduction of the TiO_2_ phase at 1050 °C gives rise to the formation of a mixture of sub-stoichiometric Ti-oxides [13]. The occurrence of these processes was confirmed by XRD analysis (Figure 3) showing typical peaks of Ti*_n_*O_2*n*−1_ (Magneli phase). In particular, the XRD patterns of the Ti-suboxide (Figure 3) indicates the occurrence of the Ti_9_O_17_ (JCPDS: 18-1405) structure at 24°–24.5°. The Ti_6_O_11_ phase (JCPDS: 18-1401) was also present, whereas the Ti_4_O_7_ (JCPDS: 18-1402) was essentially present in traces. Thus, there was a high degree of sub-stoichiometry in the Ti-oxide reduced at high temperature. No evidence of peaks of Anatase or Rutile was observed in the XRD pattern of the reduced Ti-oxide. All of the assigned reflections belong to the Magneli phase.

**Figure 4:**
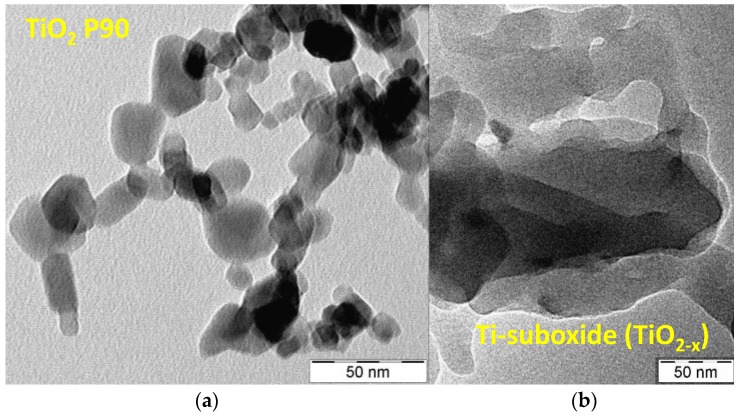
Transmission electron micrographs of a bare P90 TiO_2_ (**a**) and Ti-sub-oxide powders (**b**) used for the photoanode.

The Brunauer–Emmett–Teller (BET) surface area of P90 was around 90 m^2^/g according to the supplier, whereas the measured BET surface area of the in-house prepared Ti sub-oxide was 26 m^2^/g. This relevant difference in surface area is essentially related to the sub-oxide reduction at 1050 °C, which promotes particle sintering [13]. The morphology of the Ti-oxide materials was also investigated by TEM analysis (Figure 4a,b). The P90 Ti-oxide showed spherical particles with relatively small particle size (around 15–20 nm), whereas the Ti-suboxide, due to the high temperature treatment, showed large particle agglomerates with primary particles of about 25–50 nm in size. However, the Ti-suboxide showed high surface roughness, which is possibly associated with a large fraction of surface defects.

**Figure 5:**
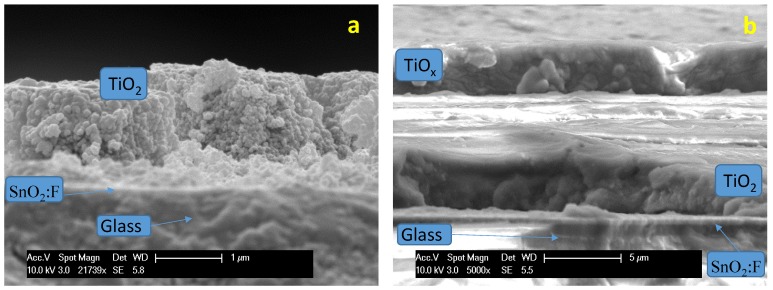
Scanning electron micrographs of a bare P90 TiO_2_ photoanode layer deposited onto a TCO substrate (**a**) and a bilayer consisting of Ti sub-oxide coated onto the TiO_2_ layer (**b**). In these micrographs, the semiconductor layers appear on stepped surfaces due to the cutting procedure.

Two types of photoelectrodes were investigated. A bare TiO_2_ layer was coated onto the TCO substrate and used in the first experiments (Figure 5a). In a second set of experiments, the TiO_2_ photo-anode layer was coated with a thin titanium sub-oxide layer (Figure 5b). The cross-section of photoanodes prepared in a similar way was investigated by scanning electron microscopy. The thickness of the TiO_2_ layer ranged between two and four microns, whereas the thickness of the Ti-suboxide layer was about three microns.

### 2.4. Photo-Electrolysis Behaviour

The influence of the membrane and ionomer characteristics on the water photo-splitting performance was investigated in a photo-electrolysis single cell. As discussed above, the photo-electrolysis device consisted of an assembly between the TiO_2_ photo-anode, the membrane and platinum as cathode. In this system, the hydrogen evolution occurs at the Pt cathode that was used as working/sense electrode, whereas, the photo-anode, where O_2_ evolution occurs, was used as counter/reference electrode. Since the aim of the work was to compare the effect of the ion exchange membrane on the water photo-splitting, a Titania-based photo-anode was initially used without any modification in terms of doping, surface treatment with reaction promoters, etc., as it would be required to achieve useful conversion efficiencies. Only a thermal sintering at 450 °C of the photo-anode was carried out to achieve good adhesion of the photo-electrode film to the substrate and proper continuity of the TiO_2_ network in the electrode layer. It is pointed out that an un-doped TiO_2_ according to its wide energy gap can absorb just a small fraction of the solar spectrum in the UV region. This system thus generates low photocurrents; however, at the same time, the high energy gap allows for achieving large spontaneous photo-voltage.

According to the sketch presented in Figure 1, being the TiO_2_ semiconductor (SC) brought in contact with the water film that is embedded into the ionomer and membrane electrolyte, a depletion of majority charge carriers (n-type in TiO_2_) across the interface occurs due to the difference in the chemical potentials of the two phases [15,16]. This results in a band bending phenomena in the semiconductor near the interface. The illumination of the semiconductor–electrolyte interface causes a modification of the electrode potential as a consequence of the generation of electron–hole pairs, which are thus separated by the effect of the electric field across the depletion region [15,16]. At the open circuit (OCP), the illumination causes an accumulation of photo-generated charge carriers across the depletion region with a corresponding upward band bending producing a spontaneous photo-voltage [16]. The band bending can be increased by polarising the electrode-electrolyte interface (Figure 1 is showing the band bending in TiO_2_ at the short circuit under illumination).

**Figure 6:**
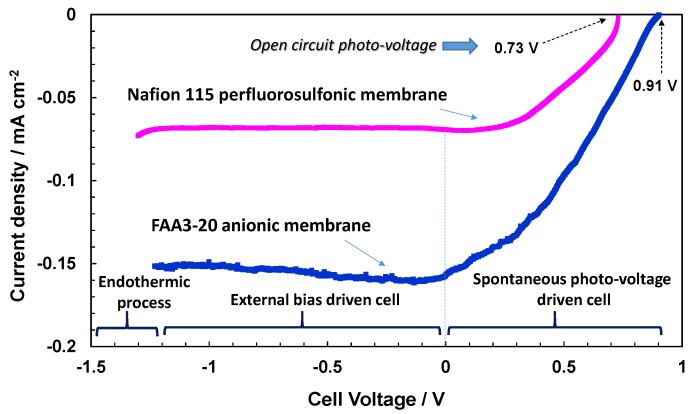
Photo-electrolysis polarisation curves under AM1.5 illumination for Nafion^®^ 115 and Fumion^®^ FAA3-20 membranes based cells consisting of a bare P90 TiO_2_ photoanode and Pt cathode.

**Figure 7:**
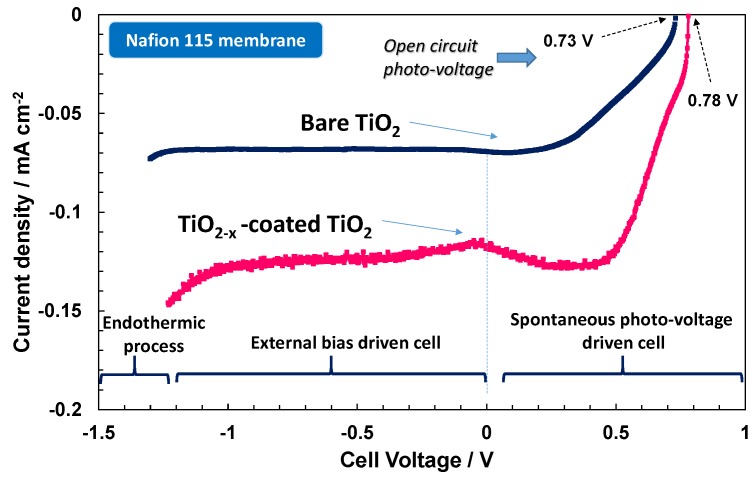
Photo-electrolysis polarisation curves under AM1.5 illumination for Nafion^®^ 115 membrane based cells equipped with a bare P90 TiO_2_ photoanode and a TiO_2−*x*_ sub-oxided coated TiO_2_ photoanode.

**Figure 8:**
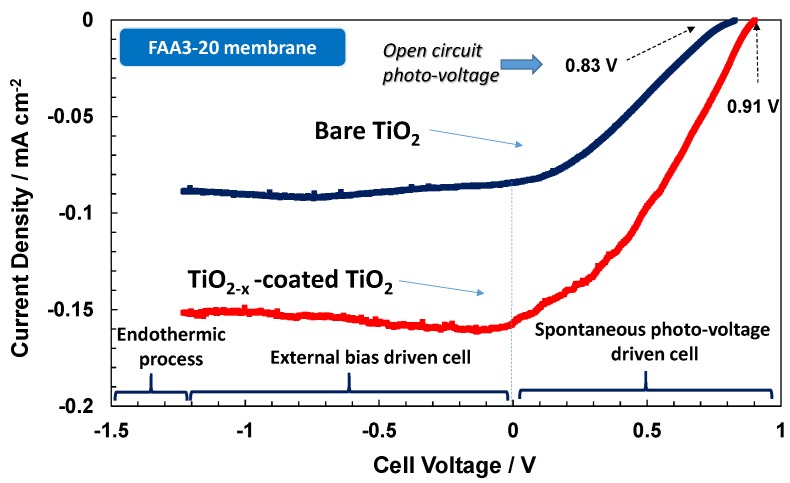
Photo-electrolysis polarisation curves under AM1.5 illumination for Fumion^®^ FAA3-20 membrane based cells equipped with a bare P90 TiO_2_ photoanode and a TiO_2−*x*_ sub-oxided coated TiO_2_ photoanode.

Accordingly, the photo-electrochemical behaviour of the electrode-polymer electrolyte membrane junction is characterised by a specific photocurrent-photo-voltage (I–V) profile that is very similar to a photodiode characteristic (Figure 6, Figure 7 and Figure 8). The spontaneous photo-voltage drives the water photo-splitting process up to the short circuit (short circuit photocurrent, Isc). No hydrogen evolution occurs at the OCP, and the observed open circuit photo-voltage represents an electromotive force for the water photo-splitting process; whereas, the spontaneous hydrogen production is, in general, maximised at the short circuit (cell potential equal to zero in Figure 6, Figure 7 and Figure 8). The potential region between the OCP and Isc is characterised by negative variation of the free energy, ∆*G*, corresponding to a spontaneous hydrogen evolution and concomitant production of electricity (positive potential region when the cathode is used as working/sense electrode) [15]. Under such conditions, the Fermi level in the semiconductor is higher in potential energy than the Fermi level in the electrolyte. This difference is the spontaneous photo-voltage [15]. The production of electricity is null at the Isc where the Fermi levels in the semiconductor and the electrolyte are the same. The polarisation range between the OCP and Isc is also called photo-voltage driven region and the photo-electrolysis cell operating under such conditions is assisted only by the solar radiation. If an external bias is applied the band banding in the semiconductor increases and the photocurrent can persist or may increase further. In this case the cell voltage is negative because the cathode, as working electrode, and its associated Fermi level in the electrolyte is higher in terms of potential energy with respect to the Fermi level in the photo-anode. Above the reversible potential (1.23 V) for water splitting, the endothermic water electrolysis occurs also in the dark. The potential region between the Isc and the reversible potential is the bias-assisted photo-electrolysis region where most of the photo-electrolysis semiconductors characterised by small band gap or un-appropriate band levels usually operate.

The I–V characteristics of the photo-electrolysis cells under investigation are shown in Figure 6, Figure 7 and Figure 8. The I–V response is reported under illumination only; the dark current was quite low and similar in all cases. As reported elsewhere [14], for a large band gap n-type TiO_2_ semiconductor, the equilibrium concentration of holes is very low. Accordingly, the anodic dark current that derives from the diffusion of holes towards the surface is also low.

The photo-electrolytic characteristics for the bare TiO_2_ photo-anode combined with proton exchange Nafion^®^ 115 and anion exchange Fumion^®^ FAA3-20 polymer electrolyte membranes are shown in Figure 6. Upon illumination (100 mW cm^−2^), the spontaneous photo-voltage registered for the Nafion^®^-based cell is about 0.73 V, where it reaches 0.91 V with the alkaline membrane. More relevant is the increase of photocurrent with the alkaline system in the overall potential region. The short circuit photocurrent recorded with the anionic membrane is about twice that observed with Nafion^®^. The higher slope of the photo-electrolytic characteristic recorded with the anionic membrane in the photo-voltage-driven region is indicative of a better fill factor corresponding to a lower occurrence of recombination phenomena [15,16,17,18,19,20,21,22]. Thus, the minority charge carriers (holes for the n-type TiO_2_) are more easily transferred to the electrolyte under alkaline conditions. This aspect may be related to the larger concentration of OH species under alkaline conditions that are involved in the intermediate steps of the oxygen evolution process [15,16]. A large concentration of these species at the interface favours their adsorption on the photo-anode surface favouring the capture of holes by the adsorbed OH species with respect to the recombination with photo-generated electrons inside the semiconductor.

The large spontaneous photo-voltage recorded for the present electrolytic cells is associated with a relevant upper band bending occurring at the TiO_2_ semiconductor upon illumination. This produces the onset of photocurrents at potentials quite negative with respect to the equilibrium potential of water splitting, which is the same in acid and alkaline electrolytes, i.e., 1.23 V at 25 °C. Thus, the energy required for water splitting is provided by the illumination and the generated high energy holes (Figure 1 bottom) can oxidise water. Accordingly, water splitting can occur under conditions more favourably than at the equilibrium, and electricity is also generated together with hydrogen at positive cell potentials. The protonic or anionic membranes do not affect this mechanism but they influence the charge transfer at the photo-anode–electrolyte interface that must compete with the recombination processes inside the semiconductor. Besides the above-discussed mechanism, another phenomenon may occur in the photo-electrolysis device that can explain the large spontaneous photo-voltages observed here. However, specific efforts were adopted to avoid exposure to air of the water filled to the cell, since the cell itself is not gastight (the cell is designed to favour gas escape), and it is not excluded that some air infiltration may have occurred during this procedure and when the cell was operated. This would mean that part of the large spontaneous photo-voltage observed here could also derive from a shift of the Fermi level of the Pt electrode to positive values associated with the adsorption of oxygen species on Pt.

In principle, the photo-voltage is caused by a shift of the Fermi level of TiO_2_ upon illumination from its rest potential in the dark, but the Fermi level positioning in the dark is determined by a specific interaction between the semiconductor surface states and the electrolyte. In our case, the electrolyte is composed of pure water in combination with a solid polymer electrolyte. The thermodynamic basis for hydrogen production relies on the fact that the conduction band edge of TiO_2_ is higher in energy or negative in potential with respect to RHE under the present experimental conditions (Figure 1 bottom). On the other hand, the measured cell voltage under illumination results from the difference between the cathode potential and the anode potential. The anode potential is affected by the interaction between the semiconductor surface states and the electrolyte whereas the cathode potential may also be affected by the adsorption of traces of oxygen gas molecules on the Pt surface.

The oxygen evolution process occurring at the TiO_2_ surface is widely considered to be the rate determining step for the photo-electrolysis configuration under study in both acidic and alkaline systems [17,18,19,20,21,22]. Light harvesting produces the intrinsic ionization of the titania semiconductor, which leads to the formation of holes in the valence band and electrons in the conduction band provided that the energy of photons (*hν*) is larger than the energy gap:

2*hν* → 2e^−^ + 2h^+^; 2h^+^ + H_2_O → ½ O_2_ + 2H^+^; 2H^+^ + 2e^−^ → H_2_ (1)

The electric field at the electrode–electrolyte interface avoids recombination of the photo-generated charge carriers allowing the light-induced electron-hole pairs to produce the splitting of water into oxygen and hydrogen at the photo-anode and photocathode–electrolyte interface. Besides influencing the charge transfer at the interface, a different type of electrolyte can favour the formation of electronic levels at the surface causing a pinning of the Fermi level [23]. Accordingly, the difference in the observed photo-voltages for acidic and alkaline environment is associated with these two effects i.e., surface states and charge transfer kinetics.

To get more insights on the surface effects and the influence of the different electrolytes on the adsorption properties of OH species, the TiO_2_ photo-anode was coated with a thin Titanium sub-oxide layer. The presence of the sub-stoichiometric TiO_2−*x*_ species on the photo-anode surface promotes significantly the adsorption of OH species. These suboxides have been identified as promoters for the oxygen evolution in acidic environment [13]. Figure 7 shows a small enhancement of photo-voltage for the Nafion^®^ based photo-electrolysis cell when TiO_2−*x*_ is coated on the TiO_2_ photo-anode and a significant increase of photocurrent in the entire range, especially at potentials close to the reversible potential (−1.23 V). This provides a clear indication that OH adsorption is a rate determining step in the photo-electrolytic water splitting in acidic environment being this process promoted by the presence of sub-stoichiometric oxides. TiO_2−*x*_ surface states favour the adsorption of the oxygen species on the surface to saturate the defective sites. The presence of a wide coverage of OH species on the electrode surface favours the hole transfer to the electrolyte while avoiding the recombination phenomena [22,23,24,25]. The positive effect is observed in terms of both photo-voltage and photocurrent. The adsorption of OH species possibly shifts the flat-band potential producing an increase of the photo-voltage. In any case, the performance of the TiO_2−*x*_-coated TiO_2_ in acidic environment does not reach that of bare TiO_2_ in alkaline environment both in terms of OCP and photocurrent.

Curiously, when the TiO_2−*x*_-coated TiO_2_ photoanode is used in the presence of the anionic system (Figure 8), a strong decrease of performance both in terms of photo-voltage and photocurrent is observed. It appears that the large concentration of OH species in the alkaline environment causes a strong adsorption on the Ti sub-oxide surface, and this hinders the desorption of oxygen species to give rise to oxygen gas evolution. It is well known that in electro-catalytic reactions, the binding energy between the adsorbed species and the electrocatalyst surface should not be neither strong nor weak but intermediate to maximise the reaction rate [7,13]. Otherwise, the rate of adsorption or desorption becomes rate determining if the bond strength is weak or strong, respectively [7,13].

The presence of widely distributed electronic levels in the sub-stoichiometric oxide surface causes recombination and trapping effects for the photo-generated carriers if the oxygen species can not be easily desorbed after the capture of photo-generated holes, thus determining low photocurrent.

The present results clearly show how the type of electrolyte can drastically influence the choice of surface promoters in photo-electrocatalysis and the performance achievable with different photo-electrode systems.

**Figure 9:**
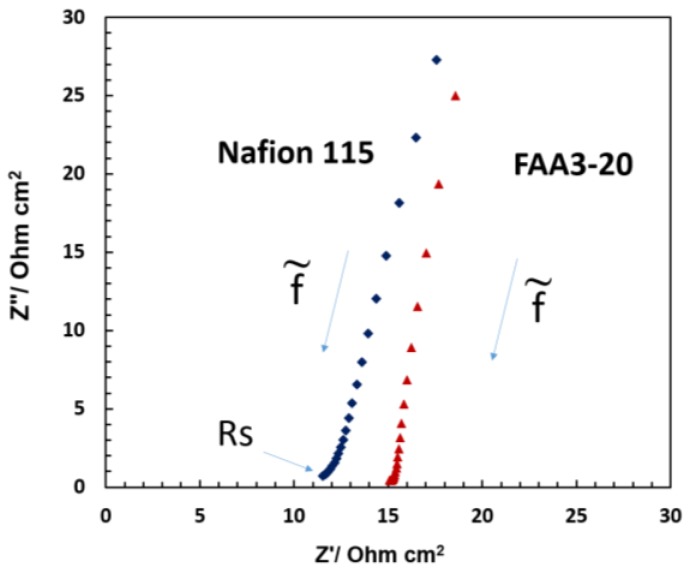
AC impedance spectra under AM1.5 illumination at open circuit potential for Nafion^®^ 115 and Fumion^®^ FAA3-20 membrane based cells equipped with a bare P90 TiO_2_ photo-anode.

Ac-impedance analysis (Figure 9) was carried out to understand if there was some relevant ohmic limitation associated with the different membranes used in the photo-electrolysis cells. The series resistance or the high frequency intercept in the Nyquist plot observed for the various systems under illumination was generally lower than 15 Ohm cm^2^. Thus, this should not produce any relevant potential loss since the photocurrent measured in the photo-electrolysis device is relatively small. However, polarization resistance associated with both the charge transfer at the electrode–electrolyte interface and the charge transport in the space charge region appears dominant since no complete semicircle is observed. The impedance profile in the Nyquist plot is mainly capacitive and the high frequency intercept shifts to larger values for the alkaline membrane. The Nafion^®^ based cell shows slightly lower series resistance due to the better conductivity even if the Nafion^®^ 115 membrane thickness is much larger than the FAA3-20 membrane. Most of the contribution to the series resistance is generally due to the TCO substrate even if the membrane, interface and cell design can also have an impact on the ohmic properties of the cell.

**Figure 10:**
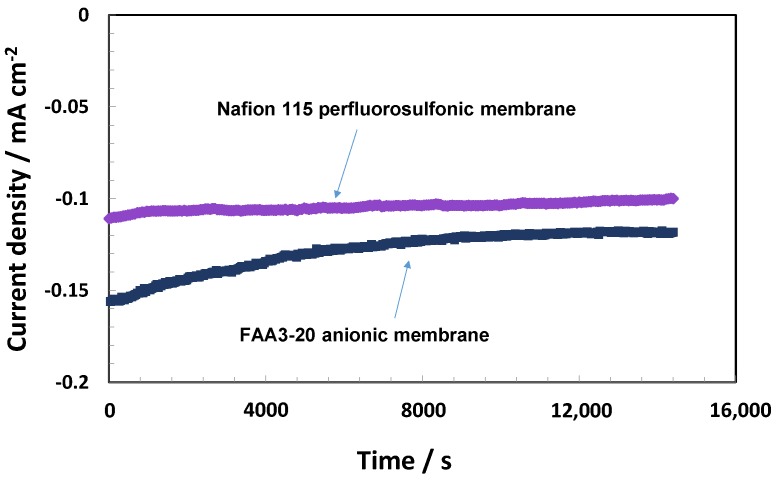
Chrono-amperometric curves under AM1.5 illumination at 0 V for Nafion^®^ 115 and Fumion^®^ FAA3-20 membrane-based cells equipped with a TiO_2−*x*_ sub-oxide coated TiO_2_ and bare P90 TiO_2_ photo-anodes, respectively.

The photo-electrolysis devices were also investigated in terms of time studies (Figure 10). For these studies, only the cells providing the best performances during the initial experiments, i.e., the bare TiO_2_ in combination with the anionic membrane and the Ti sub-oxide coated-TiO_2_ combined with the protonic membrane, were selected. Chrono-amperomentric curves (Figure 10) were carried out for both anionic and protonic devices for 4 h at the short circuit (0 V). 

**Figure 11:**
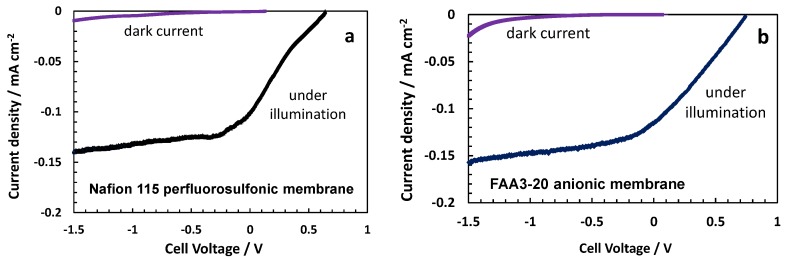
Photo-electrolysis polarisation curves carried out after 4 h operation at the short circuit under AM1.5 illumination and in the dark for Nafion^®^ 115 (**a**) and Fumion^®^ FAA3-20 (**b**) membrane-based cells equipped with a TiO_2−*x*_ sub-oxided coated TiO_2_ photoanode and a bare P90 TiO_2_ photoanode, respectively.

A gradual decrease of photocurrent with time was observed for both configurations even if the Nafion^®^-based system appeared more stable (Figure 10). After the time study, photoelectrochemical polarisations were repeated in the dark and under illumination (Figure 11). The open circuit photovoltage decreased by about 170 and 140 mV in the case of the anionic and protonic cells, respectively, with respect to the initial measurements. However, the photocurrent decay was about 25% and 11% for the anionic and protonic cells, respectively, compared to the initial values. The curves registered in the dark showed a positive voltage of about 130 mV and 97 mV for the protonic and anionic cells, respectively (Figure 11). However, the dark current was negligible in the photovoltage-driven region, increasing rapidly close to the reversible potential. This increase of dark current in the bias-assisted region close to the reversible potential was more evident for the anionic system compared to the protonic system.

Several hypotheses are formulated for the photocurrent and photo-voltage decay observed after the time studies. However, we have used deaerated water, since this is filled in the cell by a syringe system, water is necessarily exposed to the air. Thus, we do not exclude that some traces of dissolved oxygen may be presented in water. This may have possibly caused a depolarisation of the cathode by shifting the Fermi level to higher potentials. Such effect may explain the loss of photo-voltage after prolonged operation since the residual oxygen at the Pt electrode is consumed by the produced hydrogen. The decrease of photocurrent could be related to mass transport limitations for the evolution of the gas molecules inside the device or to some modification of the electrode–electrolyte interface. However, the performance decay was almost reversible in both cases since the photo-electrolysis cells re-gained the initial performance when the electrochemical operation under illumination was stopped for some hours. In this regard, we think that device design should be improved to address these recoverable losses.

The information gained from this study, using TiO_2_ as model system, and the selected membranes can be useful in developing photo-electrolysis cells based on smaller energy gap semiconductors than TiO_2_, thus providing a relevant increase of water splitting efficiency.

## 3. Materials and Methods

A commercial TiO_2_ powder, Degussa P90, was used for preparing the photo-anode. The Ti-suboxide (TiO_2−*x*_), used as surface promoter was prepared according to Ref. [13]. The thermal treatment was designed to achieve a sub-oxide phase while avoiding growth of catalyst particles. The eventual occurrence of hot spots, often causing particle sintering, was controlled by an infrared camera [26].

Briefly, the ceramic powders were prepared from TiCl_4_ by complexation of Ti ions using EDTA (Aldrich, Milan, Italy) as chelating agent, and successive decomposition by hydrogen peroxide of the formed complex to produce an amorphous oxide. A high temperature reduction (1050 °C) of the amorphous oxide was carried out by using diluted hydrogen (10% H_2_ in Ar).

Structural characterization of the Ti-oxide powders was carried out by X-ray diffraction using a Panalytical X’Pert powder diffractometer (Philips, Eindhoven, The Netherlands) with CuKα radiation. Surface area and pore volume were investigated by BET analysis using a Micromeritics ASAP 2020 instrument (Micromeritics, Milan, Italy). Morphological characteristics of the powders were observed by Transmission electron (microscopy (TEM) measurements (FEI CM12 microscope, Eindhoven, The Netherlands).

For photoelectrolysis measurements, the TiO_2_ photo-anode was prepared by spraying solutions of TiO_2_ nanoparticles dispersed in water and isopropyl alcohol onto a transparent conductive oxide, TCO, (19 Ω/sq.) substrate (SnO_2_:F). In a second set of experiments, an additional layer formed by the Ti-suboxide was deposited onto the TiO_2_ layer. Before use, TCO glasses were treated for 5 min in an ultrasonic bath of isopropyl alcohol and rinsed with acetone. Triton X-100 was added as dispersant. The coated electrode was dried at 70 °C onto a hot plate and then annealed in an oven at 450 °C for 30 min in air. The active area was 4 cm^2^. For the cathode, the TCO substrate was coated with a drop of H_2_PtCl_6_ and dried at 70 °C onto a hot plate. It was thermally annealed at 450 °C to form a Pt black particles-based layer onto the TCO. This was used as counter electrode for hydrogen evolution.

The morphology of the photo-anode was investigated by scanning electron microscopy (FEI XL30 SFEG, Eindhoven, The Netherlands).

Commercial perfluorosulfonic acid (Nafion^®^ 115) and quaternary ammonium-based anionic (Fumatech^®^, Fumasep^®^ FAA3) ion exchange membranes were purified before use (a picture of the raw membranes is shown in Figure 1). Ex situ membrane characterisation was carried out according to the procedures described in Ref. [27].

Nafion^®^ 115 (Ion Power, equivalent weight, EW, 1100) of 125 μm thickness was used as proton exchange membrane polymer electrolyte. The Nafion^®^ membrane was purified with H_2_SO_4_ 0.1 M for 1 h, treated with water several times up to achieve neutral pH for the washing solution and successively boiled in water before use. In addition, 5 wt % Nafion^®^ ionomer (Ion Power, 5 wt % solution) was used as dispersion. This was deposited on the formed anode, which was dried at 120 °C for 10 min to evaporate the solvent. The ionomer dispersion was also deposited on the counter electrode.

A Fumatech^®^ FAA3 anionic membrane of 20 μm thickness was purified with 0.5 NaOH for 1 h to produce a high swelling of the membrane and successively with NaCl 0.5 M for 72 h at 25 °C, exchanging the solution several times to replace bromide counter ions with chlorides. Thereafter, the membrane was washed in water and treated with NaOH 0.1 M at room temperature for 1 h. Finally, it was treated with water several times to achieve neutral pH for the washing solution.

The Fumatech^®^ FAA-3 dispersion was obtained by dissolving the FAA3 powder (5 wt %) in ethanol and *n*-propanol. The dispersion was deposited onto the photoanode surface and dried at 60 °C to evaporate the solvent. The coated photoelectrode was first treated with 0.1 M NaCl and thereafter with 0.1 M NaOH. The same procedure was adopted for the counter electrode.

The ionomer-coated electrodes, TiO_2_/TCO photoanode and Pt/TCO cathode were pressed together onto the membrane using an ultrathin segmented sub-gasket in between to allow for gas escape from the two cell compartments. The segmented sub-gasket was laser cut and made of the same ion conducting polymer material as the membrane. Deaerated Milli-Q^®^ water was fed to the cell through a hole drilled on the Pt/TCO counter electrode until achieving full hydration of the cell.

The electrochemical apparatus consisted of a Metrohm Autolab potentiostat/galvanostat (Utrecht, The Netherlands) equipped with a Frequency Responce Analyser (FRA). An Oriel^®^ solar simulator (Newport, Irvine, CA, USA) providing an irradiation of 100 mW cm^−2^ (AM1.5) was used. The cell was illuminated through the photo-anode glass backing. Thus, the light penetrated through the glass and the transparent TCO layer before reaching the TiO_2_-hydrated polymer-electrolyte interface. Polarisation measurements (20 mV s^−1^) under illumination (100 mW cm^−2^) were carried out in a wide potential range starting at the open circuit photovoltage (OCP), passing through the short circuit photocurrent (Isc) up to achieving the reversible potential for the endothermic water splitting (1.23 V). Ac-impedance measurements were carried out at OCP under single-sine mode by sweeping frequencies from 100 kHz to 10 mHz using 10 mV r.m.s excitation sinusoidal signal.

## 4. Conclusions

Perfluorosulfonic acid (Nafion^®^ 115) and quaternary ammonium-based anionic (Fumatech^®^ FAA3) ion exchange membranes were investigated for photo-electrolysis applications using TiO_2_ photo-anode as a model system. This allowed for getting insights about the behaviour of the different membranes both under photo-voltage-driven and bias-assisted operations. The two polymer electrolyte membranes systems were used together with the corresponding ionomer dispersion and determined the occurrence of different reaction environments since pure water only was fed to the cell. In particular, the membrane/ionomer chemistry produced a different operating pH and this influenced significantly the photo-electrolytic conversion. The anionic membrane enhanced the performance of the TiO_2_ semiconductor compared to the Nafion^®^ membrane as a consequence of the increased concentration of hydroxides with enhanced adsorption of OH species that promote the oxygen evolution reaction. Interestingly, this was not the case of the Ti sub-oxide coated TiO_2_ in alkaline environment. Such phenomenon was explained with the strong adsorption of OH species in alkaline environment to saturate surface defects in the sub-stochiometric oxide. Thus, strongly bonded surface oxygen species occurred, which were not easily desorbed. Such species could not acquire additional photo-generated minority carriers. These were very likely recombining with photo-generated electrons inside the semiconductor bulk. On the contrary, since the adsorption of OH species on stoichiometric TiO_2_ is very labile in the acidic environment, the increased adsorption strength at the sub-oxide surface sites played a favourable role for that oxygen evolution in the presence of Nafion. The results clearly demonstrate that the photocurrent characteristics are strongly dependent on both properties and polymer electrolyte membrane photo-anode. Membranes can be tailored to modulate electronic levels at the interface in order to achieve the best properties to match between the semiconductor band gap edges and the redox levels in the electrolyte. This may be especially useful in the case of smaller energy gap semiconductors that can harvest a larger fraction of the solar radiation than TiO_2_, thus boosting water splitting efficiency.

## Figures and Tables

**Table 1:** Ex situ characterisation data of the Fumatech^®^ anionic FAA3-20 membrane compared to Nafion^®^ 115.

Membrane Acronym	Unit	Fumasep^®^ FAA3-20	Nafion^®^-115
Trade name	–	Fumion^®^	Nafion^®^
		ionomer	ionomer
Polymer type	–	Polyaromate	Perfluorosulfonic acid
Conductivity type	–	Anion exchange material	Proton exchange material
Terminal ionic groups	–	Quaternary ammonium group	Sulfonic acid group
EW (theoretical)	g/mol	555	1100
IEC (exp.)	mmol/g	1.8	0.9
Thickness (dry)	μm	20	125
Conductivity in H_2_O at *T* = 25 °C	mS cm^−1^	6 (Cl^−^)	62 (H^+^)
		30 (OH^−^)	
